# eHive: An Artificial Intelligence workflow system for genomic analysis

**DOI:** 10.1186/1471-2105-11-240

**Published:** 2010-05-11

**Authors:** Jessica Severin, Kathryn Beal, Albert J Vilella, Stephen Fitzgerald, Michael Schuster, Leo Gordon, Abel Ureta-Vidal, Paul Flicek, Javier Herrero

**Affiliations:** 1 European Bioinformatics Institute, Wellcome Trust Genome Campus, Hinxton, Cambridge, CB10 1SD, UK; 2 RIKEN Yokohama Institute, Omics Sciences Center (OSC), 1-7-22 Suehiro-cho, Tsurumi-ku, Yokohama, Kanagawa, 230-0045, Japan; 3 Eagle Genomics Ltd., Babraham Research Campus, Cambridge CB22 3AT, UK

## Abstract

**Background:**

The Ensembl project produces updates to its comparative genomics resources with each of its several releases per year. During each release cycle approximately two weeks are allocated to generate all the genomic alignments and the protein homology predictions. The number of calculations required for this task grows approximately quadratically with the number of species. We currently support 50 species in Ensembl and we expect the number to continue to grow in the future.

**Results:**

We present eHive, a new fault tolerant distributed processing system initially designed to support comparative genomic analysis, based on blackboard systems, network distributed autonomous agents, dataflow graphs and block-branch diagrams. In the eHive system a MySQL database serves as the central blackboard and the autonomous agent, a Perl script, queries the system and runs jobs as required. The system allows us to define dataflow and branching rules to suit all our production pipelines. We describe the implementation of three pipelines: (1) pairwise whole genome alignments, (2) multiple whole genome alignments and (3) gene trees with protein homology inference. Finally, we show the efficiency of the system in real case scenarios.

**Conclusions:**

eHive allows us to produce computationally demanding results in a reliable and efficient way with minimal supervision and high throughput. Further documentation is available at: http://www.ensembl.org/info/docs/eHive/.

## Background

The Ensembl project provides an integrated system for the annotation of chordate genomes and the management of genome information [1]. Ensembl produces several releases per year. In every release, data updates are provided for recently sequenced species, for those species with new assemblies and when additional information is available. For instance, a new set of RNA-seq data can be used to refine the structure of the genes or other features. The data is provided through the Ensembl Genome Browser (http://www.ensembl.org), a Perl API, via direct querying of the underlying databases or via Biomart, a data-mining tool [2]. The same public Perl API is used by both the web server to fetch the data from the database and the project members themselves for accessing data, analysis and storing the results of the analyses.

Each Ensembl release requires the coordination of several analysis teams involved in the process from gene annotation to the final display of all data on the web. The Ensembl Comparative Genomics group has approximately two weeks to create the comparative genomics data. The number of species included in Ensembl grows with nearly every release. For instance, Ensembl release 56 (September 2009) includes 50 genomes for which genomic alignments and protein homologies predictions are produced. Although most of the jobs required to create the comparative genomics resources are small, some of the steps in the pipeline involve comparisons between all possible pairs of species in Ensembl, and the amount of computational effort required for some of these steps grows almost quadratically with the number of species. As there is usually at least one new species or an updated assembly in every release, this means that the most expensive calculations, the ones involving all species, are re-run for every release.

In order to solve these scalability challenges as the number of species grows, we have designed a production system able to process huge amounts of small jobs and run autonomously with minimal manual intervention. This new fault tolerant distributed processing system is based on several concepts common in Artificial Intelligence (AI), namely (a) blackboard systems, (b) network distributed autonomous agents, (c) dataflow graphs, and (d) block-branch diagrams. The basic goals of the system are (a) to reduce the overhead of individual job processing, (b) increase the maximum number of jobs that can be in-flight at any one time, (c) provide fault tolerance, and (d) allow concurrent runtime processing capable of implementing complex algorithms with branching and looping as part of the work flow.

We call our production system eHive and use it to manage and control the comparative genomics workflow and job scheduling requirements. eHive is fully integrated with and makes extensive reuse of the software modules available in the family of Ensembl Perl APIs.

### Artificial intelligence system design

The design of the eHive system is specifically inspired by the descriptions of artificial life from sources such as Reynolds [3] and incorporates several specific AI and workflow graph theories described below. In artificial life systems such as eHive, intelligent agents are created with a basic set of behavior rules that depend in part on the behavior of the agents closest to them. This programming creates a cooperativity among the agents and results in any given agent having the greatest behavioral effect on the agents closest to it in the system and little or no effect on an agent far away.

In these models, the system often exhibits global characteristics resulting from agents working together locally without explicit need for communication with all of the other agents working simultaneously in the system. These global characteristics, which are not explicitly programmed into the system, are termed "emergent behavior" and are a critical advantage of the eHive over other job scheduling methods. The rules for the eHive (and, indeed, the name of the system) are modelled on a living, active "honeybee" hive. In sort, we can summarize the rules for each agent (i.e. "worker bee") as follows:

• Identify an available and appropriate work object

• Ensure any work objects arising from completion of the current work object can be identified by follow-on agents

• Work optimally through out the lifespan

• Die gracefully

### Problem solving model

The key concept to enable the eHive system is the blackboard model of problem solving [4]. Briefly, a blackboard model provides a means for knowledge sources (i.e. "workers") to generate and store partial solutions to a problem without direct communication between the workers. Instead, the workers read and write information to a central globally accessible database (the "blackboard"). In a blackboard model, the choice of worker type is based on the solution state at any given time. For example, if no tasks of type B can begin until at least 25% of tasks of type A have been completed, no type B workers will be created until the blackboard shows the completion of at least 25% of the type A tasks. The strength of the blackboard model is that each part of the problem is solved by a specialist application in an incremental and opportunistic way which responds to the ongoing changes in the solution state. In combination with the local model of behavior, a global pattern of system control emerges without the explicit need for a central controller to analyze the entire blackboard and make detailed decisions.

#### Network distributed autonomous agents

Within the eHive a collection of agents actually do the work. These agents can be classified as both collaborative agents and reactive agents using the terminology of Nwana [5]. Collaborative agents generally work autonomously and may be required to cooperate directly with other agents in the system. In our application, the agents are distributed across the compute cluster and communicate only with the blackboard. Reactive agents respond to the state of the environment (in our case the state of the blackboard) and thus may change their behavior based on the progression of the partial solution recorded on the blackboard. When their interactions are viewed globally, complex behavior patterns emerge from systems with reactive agents.

#### Control structures

The two major control structures used for algorithm development within the eHive system are data flow graphs and block branch diagrams. Data flow graphs represent data dependencies among a number of operations required to solve a problem. The block branch diagram (BBD) is a common aspect of object oriented software design. For each function, the BBD represents the control structure, input and output parameters, required data and dependent functions.

## Results

Scheduling systems for large compute clusters are generally based on the idea of a central job queue and a centralized job manager. Cluster nodes are "dumb" and need to be given explicit instructions for each and every job they will need to execute. This creates a bottleneck in the central controller. Many of these systems have a latency of several seconds between the job submission and its execution and most are designed around the idea that jobs will run for an hour or more. They are not designed for handling 100 million jobs that run for only a few seconds each. To manage this increased job queuing overhead, applications with large numbers of short jobs often require another system on top of the job scheduler to "batch" jobs so that they can match the parameters of the job scheduler.

The basic function of the eHive system is to move the job scheduling function away from the center, but still to retain the ability to monitor and track jobs. This is accomplished using the AI systems described above to create a behavior model based on an analogy of a honeybee queen in a hive and her worker bees.

In the eHive, the jobs are no longer "scheduled" by a central authority, but each autonomous worker created by the queen now employs an algorithm of "job selection and creation" based on observing the central Blackboard with different levels of granularity. The result is a hive behavior system with a very small (3 msec) job overhead, fault tolerance and an ability to efficiently utilize CPU clusters with more than 1000 processors.

Additionally, and significantly different than a central job scheduler, the eHive system is a full algorithm development platform. It implements the concept of dataflow as well as branching and looping. It allows any algorithm that can be described in block-branch notation to be implemented as eHive processes including serial or parallel algorithms.

## Implementation

**Figure 1:**
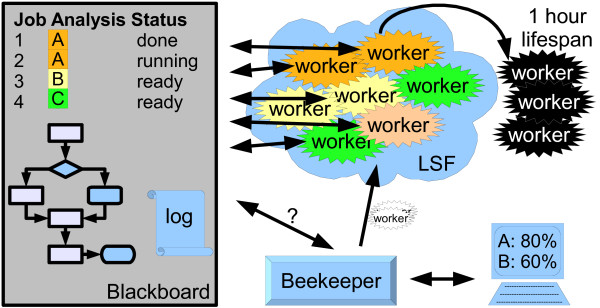
**eHive system overview**. The eHive system is based on a Blackboard System implemented as a MySQL database. It contains a list of all the jobs to run as well as dataflow and branching rules. The operator monitors and controls the system using a program called beekeeper. It connects to the blackboard and creates workers as required. Workers run in a queuing environment, typically LSF. They run jobs for a particular analysis until no more jobs are available or they reach the end of their one hour lifespan. The eHive also keeps track of the throughput of the pipeline as it runs.

The Blackboard System of the eHive contains both the list of all the jobs to run and the dataflow and branching rules. The agents connect to the Blackboard to find new jobs and to post newly created jobs back, thereby facilitating indirect communication with other workers. Normally, each job has to fetch some input data from a shared resource like a network file system or a database. Also, each job will typically store the results in a common database, which would commonly create a bottleneck if too many agents were running at once. Thus, the system is designed to control the total number of simultaneous agents. Ideally, each agent runs in a farm environment with a job queuing system (Figure 1). Although Load Sharing Facility (LSF) [6] is used by default in our implementation, the eHive has been successfully tested with Sun Grid Engine (SGE) [7] and the Portable Batch System (PBS) [8].

### Blackboard System: the eHive database

**Figure 2:**
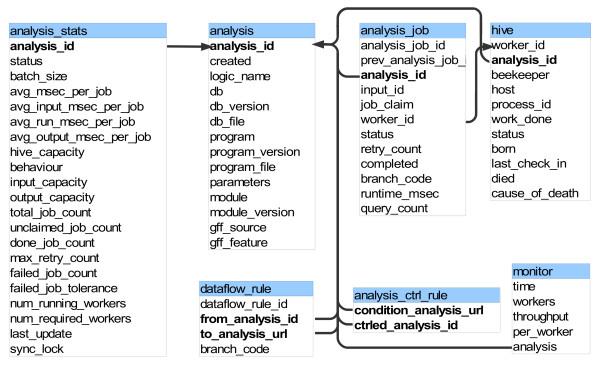
**eHive database schema**.

The Blackboard is implemented as a MySQL database. All the Autonomous Agents have read and write access to this database. The main system relies on the following tables (Figure 2):

• **hive**: The hive table keeps track of all the autonomous agents. It contains information about their status, the number of jobs they have processed, when they were born, as well as the time and cause of death for dead agents.

• **analysis**: All of the different types of analyses are listed in the analysis table. It includes two main values: the module and the parameters columns. The module corresponds to the name of a Perl module in the production pipeline. It must implement the following methods: fetch_input, run and write_output, which are run in this order by the autonomous agents. Configuration parameters for this module are stored in the parameters column.

• **analysis_stats**: This table provides high level statistics on the state of the analysis and its jobs. It also defines the behavior of the workers.

• **analysis_job**: Each job is represented by one entry in this table. There is a link to the analysis table and both the input_id and the parameters value from the analysis table define the specific options for this job. This provides sufficient information to run the job. Also, this table stores information about the status of each job, the timestamp when the job was started, a link to the hive table to record which worker/agent processed this job. Upon job completion, the actual total runtime of the job is also recorded.

• **analysis_ctrl_rule**: This table sets control rules (priorities) between the analyses. Ultimately, it defines the order in which the jobs must be run. The structure is very simple as it only contains two columns: condition_analysis_url and ctrled_analysis_id. The condition_analysis_url defines the analysis that must be done before starting the analysis referred to in the ctrled_analysis_id column.

• **dataflow_rule**: This table defines the fate of the output of a job. Upon completion, a job has the opportunity to create new jobs based on its result. This table can be used to connect the output of an analysis to the input of another analysis. Depending on the analysis, each job can create 0, 1 or several new jobs at completion. Both this and the previous table define the dataflow graph for the pipeline.

• **monitor**: This table is used to log the number of running workers and the current throughput of the eHive.

### Autonomous Agents: the Workers

Each worker is a Perl script that runs autonomously in a computational cluster environment. Its mission is to grab and run jobs posted on the eHive blackboard and to optionally post new jobs back. The only information given to a worker when it is launched is the URL for the location of the blackboard database. The successful birth of a worker results in the worker registering itself on the blackboard. This can only happen if the code to run the worker is accessible and functional and the blackboard is reachable. These checks ensure a high level of fault tolerance even before starting to run the jobs. Therefore, most pipeline failures occur before work happens, with very few problems that arise mid-run. Then, the worker must mature before being allowed to grab any job. During this maturity process, the worker chooses a particular analysis task as described below.

Once the worker is alive and registered with the central blackboard, the worker queries the analysis, analysis_stats, and hive tables to get a high-level summary of all the different processing nodes in the system, the numbers of current workers registered to each analysis and the amount of pending work. Next, the worker selects an analysis, registers this selection with the blackboard, and then dynamically loads the code module needed to run this analysis. We have taken advantage of the runtime code loading abilities of Perl in implementing this feature. Although the current implementation requires the code to be pre-distributed on the nodes, Perl also allows the code to be streamed from the blackboard database as text and loaded dynamically with an eval statement. Once the worker has dynamically loaded the processing module, it can run the jobs. The worker then proceeds to grab jobs from the blackboard. Once a job is finished it may post new jobs back to the blackboard and thus create more work for other workers.

Each job is broken down into 6 distinct stages (READY, CLAIMED, GET_INPUT, RUN, WRITE_OUTPUT, DONE) which occur in order. As the worker runs each job through the processing module, it reports back to the blackboard its current stage. Only once all steps are completed and the processing module has not reported any errors, will the worker set the status of the job to "DONE". In addition each job records which worker (worker_id) is running it and when it started processing. This information provides the system the ability to flag jobs that are suspected of being stalled or having failed in an uncontrolled manner providing the second level of fault tolerance within the system. These jobs can be queried by the operator to figure out what went wrong with the data or the algorithm.

Just like a worker bee, the agent is very efficient once it is an adult but it has a limited life span. By default each worker is given a life span of 1 hour. Although this specific time is due to the configuration of our shared cluster, it is typical for a batch queuing system to penalize users running long jobs. Thus, the life span limit permits a fair use of the shared computational resources and results in the eHive system taking maximum advantage of the compute farm. Each worker is allowed to process as many jobs as possible within that hour before dying, but naturally the worker is allowed to complete any job it has started.

### Dataflow graph

The general flow of work through the system is that of a cascade. The workflow graph consists of analysis nodes and dataflow rules as edges. In addition, control rules are used to define dependencies between the analyses. This can be used to force a particular analysis to wait until all its dependent analyses have finished processing all their jobs. This is important since in many algorithms there are some steps which can be run in parallel, but there are other steps which must be run sequentially. A typical workflow has one analysis at the start and a handful of jobs are defined. These jobs will create a cascade of jobs and flow them out into the next analysis processes.

The workflow graph is allowed to be fully dynamic and no restrictions are placed on the developer to implement an algorithm. For instance, workers are allowed to create analyses, dataflow rules, control rules and jobs as they run. This is possible because a worker does not need to know about the full structure of the pipeline. Instead, it only needs to be aware of the steps immediately upstream and downstream of itself in the dataflow graph.

### External control and monitoring: the beekeeper

The beekeeper serves as a console for the person running the eHive system. It is a Perl script which reports back the progress of the work and creates new workers as they are required. The workers are submitted to the LSF queue provided that the total load of the system is low enough. In normal conditions, the beekeeper is used in loop mode. The script continuously checks the system and creates workers until all the work is done. Since workers are configured with a 1 hour lifespan and created in a staggered manner, the script can sleep for 2 minutes between each loop before needing to repeat the procedure. Since the workers are self configuring, if too many workers are accidentally created, they will simply fail to mature due to lack of work.

The eHive system also includes basic monitoring functionality. Every job in the system is logged with its actual runtime so the average runtime for jobs can be estimated from the historical jobs. The hive table described above keeps track of each worker and their throughput and the analysis_stats table maintains approximate estimates of job progress and average runtime within each analysis.

Additionally, in every loop, the current number of workers and throughput of the system (as the number of jobs run per second) is logged into the monitor table. These values can be used by the operator to assess the effect of increasing or decreasing the number of workers on the throughput.

### Fault tolerance

Fault tolerance is an inherent part of the system. Since jobs are processed by already running workers, most of the common faults (hardware failures, lost disk mounts, lost network connections, missing software) are all caught before a job ever leaves the database/blackboard. Jobs that fail to complete processing (hardware failure mid-run, program error) are left in an 'unfinished' state and are easily tracked and reset. Jobs are posted to the blackboard where any worker is free to grab them. Once a job is grabbed, no other worker should take the work, but they are not prevented from doing so. This freedom allows the developer to create ad-hoc "garbage collector" processes which can reset jobs that appear to be stalled. The blackboard itself does not implement any behavior or restrictions; each behavioral aspect is encapsulated in the workers and the dynamically loaded processing modules. In the current implementation, this garbage collection of failed workers and jobs is handled by the beekeeper script.

When a job fails, it will be re-run automatically up to a certain number of times. If the failure is persistent, the status of the job in the analysis job table is set to FAILED and the job is not re-run anymore. The maximum number of retries is defined in the analysis_stats table. Usually one wants all the jobs to run successfully. In some cases, though, it might be expected and acceptable to have a small number of failing jobs. The percentage of acceptable failures is defined in the analysis_stats table. If there are too many failures, the system will halt at this point. This may happen when there is a configuration problem or any other specific issue like a problem with the filesystem or a faulty cluster node. Usually, this means some manual inspection is required to solve the issue. If there is a problem with the configuration or the computing farm, the operator can reset these jobs and resume the pipeline once the problem has been fixed. If there is a problem with one or a few specific jobs because they require more memory or temporary disk space for instance, the operator can run these jobs manually under different conditions and resume the pipeline afterwards.

### Pipeline initialization

We use a loader script to feed the system with initial values. The loader script reads the parameters from a configuration file. Most of the analyses, dataflow rules and control rules are created at this time, although some are created dynamically at a later stage. Several examples are described in the next section.

After running the loader script, the operator needs to launch the beekeeper. In normal conditions the pipeline will run automatically until completion.

## Applications and Case Studies

### Pairwise alignments pipeline implementation

Aligning two genomes would be straightforward if one could run the program in one single step. Unfortunately, the size of the vertebrate genomes involves impractical memory requirements for such an option. One naive possibility would be to split the problem by chromosome, but these are still too large in many cases. For instance, chromosome 1 of the marsupial *Monodelphis domestica *is approximately 750 Mb long [9]. On the other hand, other genome assemblies are fragmented in thousands of scaffolds (e.g. the assembly of the western European hedgehog, which has thus far been sequenced to only 2× coverage, contains nearly 350000 pieces). In order to overcome this problem, we chunk the chromosomes in segments around 10 Mb in length and we group the smaller fragments to sum up the same amount of sequence. We call these objects "DNA Collections", and these allow us to run comparison alignments of each group of chromosome segments against each other in jobs of comparable size and predictable duration. 

We employ two different pipeline strategies for whole genome alignments depending on the evolutionary distance of the genomes to be aligned. For relatively closely related genomes follows the same strategy as Kent et al. [10] for obtaining pairwise alignments. In brief, each DNA collection of one species is aligned to every DNA collection of the other species resulting in so-called BlastZ-raw alignments. These pairings are chained together with the axtChain program to form longer alignments we call BlastZ-chains. The last step involves the program chainNet to find the best-in-genome alignment in one of the two species. As a general rule, we use the human or the mouse genome as the reference when defining the best-in-genome alignments.

For greater evolutionary distances, we opt for using the program BLAT [11] in translated mode instead. BLAT performs the search in all 6 possible reading frames. By using translated alignments we increase the sensitivity of the method. Initially we find a large amount of short overlapping alignments. We use the approach previously described for finding the best-in-genome ones. This strategy improves the specificity of the alignments by reducing the total number of aligned base pairs without compromising the overall coverage (Table 1).

**Table 1:** Effect of the chain-net approach on the translated BLAT alignments

Alignment	Chain&Net	Alignments	Genomic Coverage		Coding Exon Coverage	
Human-Chicken	NO	894342	39885438	1.09%	20286249	49.55%

Human-Chicken	YES	337551	39928606	1.09%	20024694	48.91%

Human-C.savignyi	NO	142111	6039918	0.16%	4227643	10.33%

Human-C.savignyi	YES	54447	5720330	0.16%	4052706	9.90%

Mouse-Tetraodon	NO	509589	20480760	0.75%	14931981	42.29%

Mouse-Tetraodon	YES	177447	19615452	0.72%	14450833	40.93%

Mouse-Xenopus	NO	2061217	29538379	1.09%	18769694	53.16%

Mouse-Xenopus	YES	239083	27926452	1.03%	18113364	51.30%

This table compares the result of the translated BLAT alignmnts before and after using the chain-net approach. While the total number of alignments is reduced to less than 40%, the coverage is only slightly reduced.

Both pipelines share the same basic structure. One can choose one or the other by simply swapping the BlastZ-raw module by the translated BLAT one. This is an example of the flexibility provided by the modularity of the pipelines implemented in the eHive system. Changing or updating one particular step of the pipeline usually requires the modification of only a single module.

**Figure 3:**
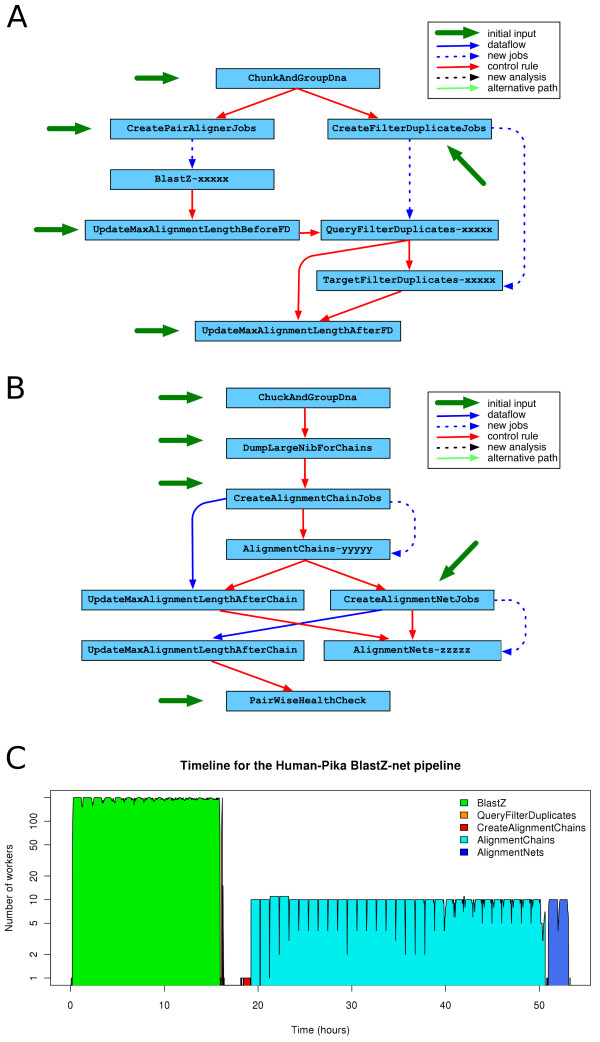
**Pairwise Alignment Pipeline**. Each analysis is represented by a blue box. The blue arrows show the flow of information from one analysis to the other, either using the dataflow rules (solid arrow) or by massive creation of new jobs as part of the analysis (dashed arrows). Red arrows represent control rules, i.e. analyses that cannot start until the previous one has finished. Black arrows show the creation of new analyses during the execution of the pipeline. Turquoise arrows show alternative paths taken when a particular job fails. The green arrows mark the initial jobs required to run the pipeline. **(A) **First part of the Pairwise alignment pipeline where we build the set of raw alignments. First, the ChunkAndGroupDna module creates one DNA Collection for each genome. CreatePairAlignerJobs and CreateFilterDuplicateJobs create the BlastZ, QueryFilterDuplicates and TargetFilterDuplicates for these DNA Collections. The BlastZ analysis runs all the BLAST [16] jobs. In order to avoid border effects due to the initial chunking process of long chromosomes, we allow partially overlapping chunks. The QueryFilterDuplicates and TargetFilterDuplicates analyses remove the duplicates and resolve the inconsistencies in the overlap between these chunks of sequences. UpdateMaxAlignmentLength analyses are needed to perform efficient ''region queries'' in a MySQL database. **(B) **Second part of the Pairwise alignment pipeline where raw alignments are chained and netted. The DumpLargeNibForChains module formats the input files for the axtChain program. The CreateAlignmentChainJobs process creates one AlignmentChains job per pair of genomic segments. The netting is performed using the same strategy: a single CreateAlignmentNetJobs job creates all the AlignmentNets jobs. Last, the PairwiseHealthCheck analysis runs a set of sanity tests on the resulting data. **(C) **Timeline of this pipeline when aligning the human and the pika genomes.

Figure 3 shows the relationship between the different analyses in the pipeline. We run the pipeline in two parts. In the first one we obtain the raw alignments (Figure 3A) between the two genomes. The second part of the pipeline (Figure 3B) consists on formatting the input, and running the axtChain and axtNet programs. Table 2 shows the number of jobs for each analysis when aligning the human and the pika genomes and Figure 3C shows the timeline for the processing of these jobs. This pipeline ran for more than two days. All the BLASTz jobs ran during the first 17 hours. The pattern of pronounced grooves is an indication of a heavy load in the compute farm: after the workers exhaust their lifespan, new workers are delayed by many other processes competing for the farm resources. Despite filtering out all non best-in-genome alignments, the final set of alignments covers all but one human genes that have a direct (1-to-1) orthologous gene in the pika genome.

**Table 2:** Jobs in the Pairwise alignment pipeline for Human-Pika

Analysis	Number of jobs	Granularity
ChunkAndGroupDna	4	2 per genome

CreatePairAlignerJobs	1	1 per pipeline

BlastZ-e1886dc	97920	As many as required

UpdateMaxAlignmentLengthBeforeFD	1	1 per pipeline

CreateFilterDuplicatesJobs	2	1 per genome

TargetFilterDuplicates-e1886dc	113	1 per human segment

QueryFilterDuplicates-e1886dc	193095	1 per pika segment

UpdateMaxAlignmentLengthAfterFD	1	1 per pipeline

DumpLargeNibForChains	2	1 per genome

CreateAlignmentChainsJobs	1	1 per pipeline

AlignmentChains-26aa1260	425698	As many as required

UpdateMaxAlignmentLengthAfterChain	1	1 per pipeline

CreateAlignmentNetsJobs	1	1 per pipeline

AlignmentNets-34de6ee1	5770	As many as required

UpdateMaxAlignmentLengthAfterNet	1	1 per pipeline

PairwiseHealthCheck	2	2 per pipeline

TOTAL	722613	

This table shows the final number of jobs run for each analysis. The last column gives a short explanation on the number of jobs for each analysis. 113 human segments correspond to the 25 chromosomes (1 to 24, X, Y and MT) and an extra 88 supercontigs not yet assembled into chromosomes. The pika genome is scattered in 193095 segments. Both AlignmentChain and AlignmentNets jobs are defined by the CreateAlignmentChainJobs and CreateAlignmentNetsJobs jobs respectively.

We usually run the first part of the pipeline and load the second part only after the first one has been successfully completed, which explains the gap in the timeline (Figure 3C). The most expensive part of this pipeline is running the axtChain software and the complexity of the chaining process grows with the number of raw alignments found in the first part of the pipeline. The break between both parts of the pipeline allows us to check that we have obtained a reasonable amount of raw alignments (up to 10 million) before attempting the chaining process. If we obtain too many raw alignments, we usually restart the pipeline using a more stringent set of parameters.

### Multiple alignments pipeline implementation

Ensembl provides global whole-genome multiple alignments. Global aligners will align a set of homologous sequences in which no major rearrangement has occurred. We need to define these sets of sequences beforehand, which we call a homology map. One of the most interesting application of whole-genome multiple alignments is the detection of conserved regions. Our pipeline is divided in these three main steps: definition of the homology map, alignment of homologous segments and detection of conservation in the alignment.

First, we obtain the orthology map between the genomes of interest using Mercator [12,13]. It uses orthologous coding exons to define blocks of orthologous segments. Each block is then aligned with Pecan [14]. Last, we use GERP [15] to detect conserved regions in the alignment. GERP uses the concept of rejected substitutions to score the conservation in every column of the alignment. Then, it uses these scores to define a set of constrained elements, which correspond to stretches of the alignment where the conservation is higher than expected by chance.

**Figure 4:**
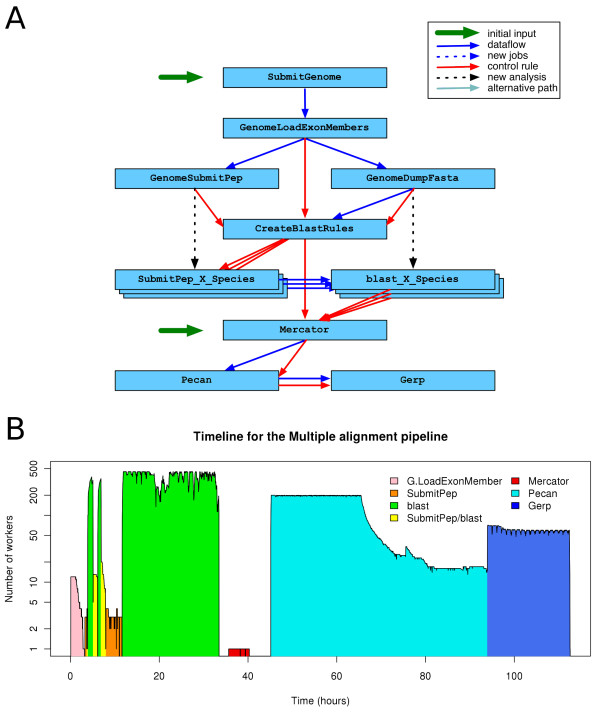
**Multiple alignments pipeline**. Colours and conventions are used as in Figure 3. **(A)**. This pipeline can be divided in 4 blocks. In the first part there is one job per species, which prepares all of the BLAST jobs. The second part (one job per coding exon) runs the BLAST jobs. In the third part, Mercator builds the orthology map using all the previous BLAST results. In the last part, each Mercator block is aligned with Pecan and GERP defines the local conservation in each alignment. In this pipeline, the SubmitPep_X_Species and blast_X_Species analyses are created dynamically by the GenomeSubmitPep and GenomeDumpFasta jobs respectively. GenomeLoadExonMembers (1 job per species) loads all the coding exons and create 1 GenomeSubmitPep and 1 GenomeDumpFasta job for each genome. The GenomeSubmitPep analysis creates 1 SubmitPep_X_Species analysis per genome and all the jobs for each of these analyses. GenomeDumpFasta creates a BLAST database for each set of coding exons and the corresponding blast_X_Species analysis. CreateBlastRules creates all the dataflow rules between the SubmitPep_X_Species and the blast_X_Species for all the other species. Mercator builds the orthology map using the results of all the previous BLAST jobs and then Pecan aligns all the orthologous genomic segments in each Mercator block. Last, the Gerp is run on each Pecan alignment. **(B) **Timeline for the 12-way Multiple alignment pipeline.

Figure 4A shows the resulting pipeline. The first part, obtaining all the exon orthology relationships for Mercator is the most complex one. The rest of the pipeline, running Pecan and GERP, is straightforward. In this example, part of the workflow graph is created dynamically. One SubmitPep and one blast analysis are required per species. These analyses are created dynamically by the GenomeSubmitPep and GenomeDumpFasta analyses respectively. They also add new control rules. The CreateBlastRules module is used to insert all the dataflow rules between the SubmitPep and blast analyses.

The actual number of jobs required to build the set of 12-way amniote multiple alignments is listed in Table 3 and Figure 4B show the corresponding timeline. Using eHive and up to 450 compute nodes, we can run over 20 million BLAST jobs [16] (including the extraction of the sequences from the database and the automatic creation of all the jobs) in approximately 1.5 days.

**Table 3:** Jobs in the Multiple alignment pipeline for 12 amniotes

Analysis	Number of jobs	Granularity
SubmitGenome	12	1 per genome

GenomeLoadExonMembers	12	1 per genome

GenomeSubmitPep	12	1 per genome

GenomeDumpFasta	12	1 per genome

CreateBlastRules	12	1 per genome

SubmitPep_*	1978219	1 per peptide

blast_*	21760409	All coding exons vs all other species

Mercator	1	1 per pipeline

Pecan	8549	1 per Mercator block

Gerp	12514	1 per Pecan alignment*

ConservationScoreHealthCheck	2	2 per pipeline

TOTAL	23759754	

This table shows the final number of jobs run for each analysis. All the SubmitPep_xxxxx and blast_yyyyy jobs have been grouped for simplicity. There are more GERP jobs than Pecan ones because alignments over 1 Mb long are split in 1 Mb segments.

### Ensembl GeneTrees pipeline implementation

The Ensembl GeneTrees are re-built for each release using all the genes from all the species available at that time [17]. We use only the longest protein as a representative of all the alternative transcripts of a gene. All the resulting proteins of one species are compared with the proteins of all the other species and the results are stored in the database. This part of the pipeline is very similar to the beginning of the previous Multiple-alignment pipeline, except that whole proteins and not only exons are aligned.

**Figure 5:**
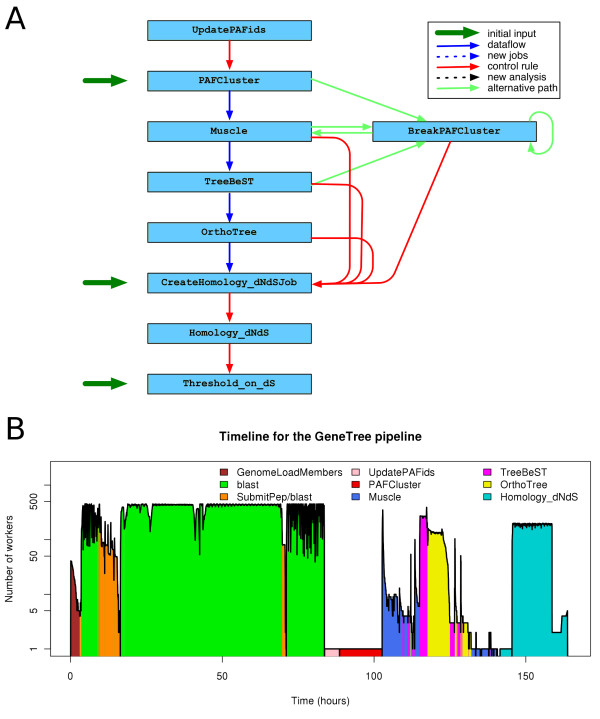
**The GeneTree pipeline**. Colours and conventions are used as in Figure 3. (**A**) The figure shows only the second half of the pipeline as the first part is very similar to the first two blocks of the Multiple alignment pipeline (panel 4A). The main difference is that we use the BLAST between the whole proteins as a block instead of splitting them in coding exons. In short, the proteins are clustered and aligned and a phylogenetic tree is built on top of each alignment. Then, the OrthoTree module calls orthologues and paralogues and the last 3 modules handle the calculation of dN/dS values for pairs of proteins. This pipeline contains alternative routes depicted in turquoise used when some particular exceptions are thrown, namely when Muscle is unable to align all the proteins in a cluster or when TreeBeST cannot infer the phylogenetic tree. This can happen when the cluster of proteins is too large. We use the BreakPAFCluster module to split these clusters in sub-groups and restart the alignment. **(B) **Timeline for the GeneTree pipeline. This figure shows the progress of the GeneTree pipeline for Ensembl release 49 (39 species). The pipeline is monitored approximately every 2 minutes. BLAST and SubmitPep jobs co-occur in one phase of the pipeline. In another phase, Muscle, TreeBeST and OrthoTree also run at the same time.

In the second part of the pipeline (Figure 5A), we build clusters using the BLAST hits, align the sequences in the cluster, infer the phylogeny from the multiple alignment using TreeBeST [18,19], extract homology relationship from the phylogenetic tree and calculate dN/dS values for pairs of proteins. This particular pipeline shows an example of the use of branching rules to handle exceptions. When the cluster of genes is too large, the multiple alignment cannot be resolved or phylogeny inference program fails, the cluster is broken in smaller parts and the pipeline continues with these.

Table 4 shows the number of jobs required to run this pipeline for 39 species. The few Muscle and TreeBeST jobs that failed were sent to the BreakPAFCluster analysis for fragmenting the corresponding cluster of proteins. The resulting clusters were sent back to Muscle as new jobs. Figure 5B shows the timeline for the whole pipeline. Running all the initial BLAST jobs took approximately 70 hours on the compute farm, with a peak of 450 concurrent jobs.

**Table 4:** Jobs in the GeneTree pipeline for Ensembl release 49

Analysis	Number of jobs	Failed jobs	Granularity
GenomeDumpFasta	39	-	1 per genome

GenomeLoadMembers	39	-	1 per genome

GenomeSubmitPep	39	-	1 per genome

CreateBlastRules	39	-	1 per genome

SubmitPep_*	682412	-	1 per peptide

blast_*	26614068	-	All vs all peptides

UpdatePAFids	1	-	1 per pipeline

PAFCluster	1	-	1 per pipeline

Muscle	26484	7	1 per genetree

BreakPAFCluster	95	-	As many as required

TreeBeST	26477	9	1 per genetree

OrthoTree	26468	-	1 per genetree

CreateHomology_dNdSJob	1	-	1 per pipeline

Homology_dNdS	3646340	1364	1 per orthologous gene pair

Threshold_on_dS	1	-	1 per pipeline

TOTAL	31022503	1380	

This table shows the final number of jobs run for each analysis during the execution of the GeneTree pipeline for 39 species. All the SubmitPep_xxxxx and blast_yyyyy jobs have been grouped for simplicity. The table also shows the number of jobs that failed. Muscle and TreeBeST jobs were recovered using the BreakPAFCluster analysis. This breaks the cluster and creates new Muscle jobs.

## Discussion

Here we describe the eHive system for large-scale genomic analysis. The eHive leverages a number of AI technologies to enable the management of hundreds of millions of compute jobs on clusters of thousands of processors. Our system is considerably more efficient that existing job scheduling systems that rely on a central job queue and a centralized job manager. We demonstrate the use of eHive's ability to manage complex pipeline workflows with several examples from the Ensembl project, including the creation of whole genome pairwise alignments, multi-species alignments and the construction of Ensembl GeneTrees.

### Flexibility

One of the main advantages of the eHive system is that the pipeline can be dynamically modified. Not only new jobs, but also new analyses, control rules and data flow rules can be created, deleted or modified as required. Also, the life cycle of the workers can be adapted to suit different needs. For instance, one could configure workers which never died and just slept until new jobs appeared. This would in effect create a system analogous to a distributed object processing system but which used a blackboard for communication, rather than a distributed object communication method.

Over time, we will want to change some parts of our pipeline. For instance, Multi-LAGAN [20] was used in our pipelines to build multiple alignments before the deployment of Pecan [14]. Similarly, our most current analysis pipeline uses Enredo [14] rather than Mercator [12], and we have recently added Ortheus to our multiple alignment pipeline to infer ancestral genomic sequences [21]. These changes are easily implemented by simply substituting or adding a step in the pipeline, with little or no changes in the rest of the modules. Typical changes that may be required in the other modules would be the addition of more information in the output of the jobs for the dataflow to work.

### Performance

Probably, the main bottleneck in the eHive system could be the access to the blackboard, i.e. to the MySQL database. Most of the stress on the database happens on the analysis_job and the analysis_stats tables. We use the InnoDB engine for these tables as it supports row-level locking. This allows several workers to update the tables in parallel. Also, to reduce the access to the analysis_stats table, the workers report about the number of jobs executed in batches. As a result, the system can easily handle more than 30 million jobs, as shown in the examples.

Some pipelines may require ad-hoc solutions to overcome other bottlenecks. For instance, in the GeneTrees pipeline, instead of storing all the protein alignments in one single table, we create one table per species to speed up the storing process. Furthermore, there is one analysis per species to run the alignments and the eHive tries to maximize the number of different alignment analyses running at once to take full advantage of this partitioning of the data.

### Scalability

The eHive system allows one to define control and dataflow rules which refer to other databases, possibly in different servers. The conditions defined in the analysis_control_rule table can be an URL to an analysis in another eHive database. Additionally, the dataflow_rule table also supports URLs for submitting jobs to other eHive systems. One can design a system where a particular analysis is managed in a secondary eHive database. The primary eHive system can submit jobs to the secondary using the dataflow rules and wait until they are resolved using the control rules. This current implementation requires independent beekeepers for each eHive system.

### Other eHive applications

It is possible to use the eHive system as a simple batch job throttling manager. This is possible with the SystemCmd module provided with the eHive system. This application of the eHive can be very useful for running thousands of commands when throttling and fault tolerance are required since simultaneously submitting all the jobs to a traditional queueing system like LSF or SGE could saturate such systems and cause failures. In such a use case, the eHive system will keep an up-to-date list of pending, running and finished jobs and the beekeeper will create new workers as needed without any manual intervention.

The eHive system is currently being adapted to applications beyond Ensembl's comparative genomics pipelines as it is especially applicable to those analyses that consist of a large number of short running jobs and those which require a cascading workflow environment for optimal resource usage.

### Alternatives to eHive

Other systems allow the users to design complex pipelines. For instance, XBaya [22] and Taverna [23,24] include a graphical interface to compose the workflow. On the other hand, they focus on using web services. Swift [25] is more similar to eHive in that both have been developed to allow running large-scale workflows using local compute resources, although remote resource can also be used.

eHive has some properties that make it especially well suited for running the pipelines aforementioned. First, it is designed to run recursive tasks. The workers are a special case of state machines who can go through one single state during their lifespan. As a result, these workers are specialized and optimized to run the same task repetitively. Second, eHive does not require a central controller. The beekeeper is only needed to create new workers. It does not assign specific tasks to any of them and does not know in which order the tasks must be solved. This is key to permit more flexibility in the system. For instance, the workers can change the structure of the workflow during the execution time. Last, eHive is fault tolerant: a job can be re-run several times after a failure before stopping the pipeline.

XBaya and Taverna use the advantage of the state machines, as each web service is specialized in running one particular task, Taverna also implements means to recover from temporary failures, but eHive is the only of these options not using a central controller and supporting dynamic pipelines. Another characteristics of eHive is the MySQL back-end to keep track of the jobs. This allows eHive to effectively handle millions of jobs. It also provides the operators with a standard and friendly way to access the data as many graphical interfaces are available for querying relational databases.

## Conclusions

One of our most important requirements is reducing the manual supervision of the pipelines. The eHive system can run in a fully automated manner and re-run jobs if they fail a reasonable number of times. Strictly speaking, the operators only need to make sure that the beekeeper is running and potentially fix any data issue if a job consistently fails. Another important feature is the ability of a worker to run more than one job, avoiding the overhead involved in submitting and scheduling a job in the queueing system. This is especially relevant when many short jobs must be run but we still want to control the process at the job level.

In conclusion, we have developed the eHive system to take full advantage of our compute farm when running our pipelines. The system is flexible enough to run virtually any pipeline, from the simplest case running as batch job throttling manager to scenarios where several eHive systems are interconnected and use different compute farms. It can easily be used for other purposes and adapted to other compute farms with little or no effort.

## Authors' contributions

JS, MS and AUV designed the system. JS developed and implemented the first versions. LG, KB, AV and JH maintain the code and have added new functionality. SF, KB, AV and JH obtained the results. PF, JS and JH designed and wrote the manuscript. All the authors read and approved the final manuscript.
